# The relationship between the preoperative plasma level of HIF-1α and clinic pathological features, prognosis in non-small cell lung cancer

**DOI:** 10.1038/srep20586

**Published:** 2016-02-08

**Authors:** Jiabei He, Ying Hu, Mingming Hu, Siyi Zhang, Baolan Li

**Affiliations:** 1 General department Beijing Chest Hospital, Capital Medical University/Beijing Tuberculosis and Thoracic Tumor Research Institute, Tongzhou District, Beijing, PR China; 2 Department of Biotechnology, Colleague of Basic Medical Sciences (CBMS), Dalian Medical University, No.9 West Section Lvshun South Road, Dalian Liaoning Province, 116044, P.R. China

## Abstract

Studies have found that hypoxia is the most common feature in all of solid tumor progression, thus it has become a central issue in tumor physiology and cancer treatment. Hypoxia-inducible factor-1α (HIF-1α) could make the tumor produce adaptive biological response to hypoxia and become more aggressive. In this paper, we used enzyme linked immune sorbent assay to detect the plasma level of HIF-1α in patients with NSCLC and healthy volunteers. The results indicated that the 5-year survival rate of patients with squamous cell carcinomas is negatively correlated with the plasma level of HIF-1α and the 5-year survival rate of patients with low level of HIF-1α is higher than those with high level of HIF-1α. The plasma level of HIF-1α in patients with NSCLC is significantly higher than healthy volunteers. There is no significant correlation between the plasma level of HIF-1α and clinical features of NSCLC patients. In a word, there is no connection between the plasma level of HIF-1α and the clinical features of NSCLC patients as well as their prognosis. In stratified analysis, the plasma level of HIF-1α in patients with squamous cell carcinoma is associated with regional lymph node status.

At Present, lung cancer is the leading cause of cancer-related deaths worldwide^1^. It is the most commonly diagnosed cancers as well as the leading cause of cancer death in males in 2012 globally. The incidence rate and mortality of lung cancer are increasing in China and some other countries of Asia and Africa. In china, the incidence rate and mortality of lung cancer are highest in malignant tumors. NSCLC is the mainly pathological type of lung cancer, accounts for about 85% of all lung cancers^2^. Surgery is an important treatment for IA–IIIA patients with NSCLC, but the recurrence and metastasis are still the leading causes of death after the resection of primary tumors^3^. The clinician needs more effective predictors or biomarkers to predict the prognosis and curative effect, so as to gain a better understanding of the patient’s clinical condition. TNM staging of the tumor and the physical condition of patients themselves are the most reliable prognostic indicator for patients with NSCLC. But even in the same stage of patients with NSCLC, there are still great differences in their overall survival (OS). No reliable indicator could be used to evaluate the therapy effect and prognosis for patients with NSCLC in the same stage exactly. Many studies have begun to look for some new biological molecules by various methods.

HIF-1α was found in the 1990s during the studies of erythropoietin expression, hypoxia is high growth-promoting factor^4^. HIFs is a heterodimer comprising HIF-α and HIF-β subunits, and there are three kinds of HIF-α, HIF-1α, HIF-2α and HIF-3α. HIF-α is the functional subunit of HIFs, and it can determine the activity of HIFs. Hydroxylation was taken place on the HIF-α by the action of prolyl hydroxylases (PHD) in normoxia, and then ubiquitination, and degraded by the proteasome rapidly^5^. Its half-life is less than 1–2 minutes. In hypoxia condition, HIF-1α’s subunit expresses stably, the accumulation of undegradative HIF-1α makes itself reach a high level and transfer to the cell nuclear. HIF-1α and the structural subunit HIF-1β form the HIF-1 heterodimeric complex, thus becoming activated HIF-1α^5^. Activated HIF-1α can mediate the transcriptional activity of the target gene by binding to the hypoxia-response element (HRE) of the target gene^6^. The functions of target genes which mediated by HIF-1α mainly includes: 1, Participate in the process of promoting erythropoiesis, thus increasing the oxygen transport of organization. 2, Promote angiogenesis and increase local oxygen flow. 3, Regulate anaerobic glycolysis, thereby adapt to local hypoxia^7^.

Previous studies had found that hypoxia was the most common feature of tumor progress including NSCLC, hypoxic zones in tumors would subsequently appear in the process of tumor growth, the reason might be that some local blood vessels unmatured cannot provide sufficient oxygen needed for tumor growth^8^. Tumor cells through a series of changes in biological characteristics to adapt to the lack of oxygen to survive and proliferation, and these adaptive changes of hypoxia make the proliferation and invasive of tumor enhance significantly^9^. Several studies had found that the possible reasons for the adaptability response may as follows: In the process of tumor growth, when the blood supply could not meet the needs of the tumor growth, HIF-1α would be activated by the hypoxia within the tumor, a series of HIF-1α dependent genes activated, regulating the biological characteristics of tumor from multiple pathways, so that the adaptive changes happened^10^,^11^.

The relationship between HIF-1α and tumor is very complex. More than one hundred kinds of target genes associated with tumor growth and metastasis can be regulated and activated by HIF-1α, most of them were related to tumor progression and metastasis, such as gene involved in angiogenesis (VEGF, PDGF, PIGF), extracellular matrix degradation gene (MMPs), metastatic gene(SDF1, CXCR4, LOX), epithelial-mesenchymal transition gene (SNAIL, SIP) and so on^12^. All genes mentioned above have been known as target genes of HIF, by promoting the proliferation of tumor cell, tumor angiogenesis, and enhancing the athletic ability of tumor cell, and improving high reactivity of tumor cell to mitogenic signals and so on, hence conducive to the growth of tumor, to adapt to the lack of oxygen^6^,^13^,^14^.

In hypoxia environment, HIF-1α can activate the adaptive response of the tumor target genes. At present, the roles of HIF-1α in tumor related are major as follows: 1) It exists in many types of cancers, its level is related to tumor invasiveness and metastatic^15^,^16^. 2) HIF-1α expression is associated with the susceptibility to chemotherapy and radiotherapy of a variety of tumors, including NSCLC^17^,^18^. A high level of HIF-1α expression can decrease the susceptibility. *In vitro* studies have found that the therapeutic effect of paclitaxel is associated with HIF-1α levels. The resistance of tumer cells to paclitaxel significantly increased when the HIF-1α overespressed^19^. It is reported that endostatin combined with radiotherapy can significantly inhibit the activity of HIF-1α in tumor cells, thus increasing the activity of antitumor angiogenesis and metastasis^20^. 3) Some researches that HIF-1α inhibitor was used for anticancer therapy have been carried out: a. Research shows that the expression of HIF-1α will be inhibited after the application of nitric oxide donor drugs, thus the drug resistance of cancer cells to anticancer drugs can be significantly suppressed^21^. In addition, we have made it clear that nitroglycerin can inhibit the role of HIF-1α. In a randomized phase II clinical trial, the researchers compared the curative effect between two kinds of different treatment methods for NSCLC patients on stage III and IV. One therapeutic method is vinorelbine and cisplatin chemotherapy combined with nitroglycerin, and the other is only vinorelbine and cisplatin chemotherapy. The results showed that nitroglycerin could significantly reduce the side effects of chemotherapy, and could increase the curative effect of chemotherapy^22^,^23^. b. Currently, some HIF-1α inhibitors such as PX-478, TX-2098 and so on, are all in the preclinical or clinical trials phase^24^,^25^. c. In addition, studies have found that some drugs that have been applied in the clinical for many years also can inhibit the role of HIF-α. For example, topotecan is a known cytotoxic drug that can destroy DNA, a few studies have found that it can directly inhibit the HIF-1α transcription significantly^26^,^27^. There are studies showed that if the patients with lung cancer recieved a low dose of topoisomerase inhibitor every day, the expression of HIF-1α in lung cancer cells can be inhibit significantly^33^. In another research *in vitro*, researchers compared the influence on tumor cells in two different therapeutic methods: bevacizumab combined with topotecan and bevacizumab received alone. The results showed that the antitumor effect of beacizumab could significantly enhance by adding the HIF-1α inhibitors topotecan^28^. d. Moreover, the efficacy of radiotherapy will be obviously improved by inhibiting the HIF-1α level in the process of radiation therapy^29^.

Currently, HIF-1α has been widely used in the evaluation of tumor in anaerobic conditions. It has been shown to be a major regulator of cell adaptation to hypoxic stress and play a critical role in oncogenesis and angiogenesis^30^. Several studies have shown that high expression of HIF-1α makes the sensitivety of many tumors to chemotherapy and radiotherapy decrease, including NSCLC^31^,^32^. In addition, it was reported that the expression of HIF-1α could predict the prognosis and therapy effect of patients with NSCLC^33^. The method used in studies of HIF-1α is mainly immunohistochemistry. The tumor samples have to be obtained from patients, however, limited the application of this method. As for the plasma level of HIF-1α and its clinical significance in NSCLC, they are rarely reported.

The above researches have demonstrated that the role of HIF-1α in tumor pathological environmental factors and a certain value of HIF-1α as a therapeutic target. Therefore, we should choose suitable patient group to apply HIF-1α inhibitors. Through testing the plasma level of HIF-1α in patients with NSCLC, maybe we can evaluate the treatment efficacy of some drugs as well as the clinical condition of patients.

## Results

### Clinical characteristics of patients

The clinical characteristics of the 100 patients and 60 cases of healthy volunteers have been presented in Table 1. There was no statistical difference in age and gender between patients and volunteers (P = 0.432, P = 0.665).

### Correlation analysis between the clinical features of NSCLC patients

The correlations between the clinical features of NSCLC patients are presented in Table 2. As can be seen clearly from the table, different histological types of patients with NSCLC have significant relationship with gender, smoking status, tumor size. There is a significant relationship between smoking status and tumor size.

### The Plasma level of HIF-1α in NSCLC patients

**Figure 1:**
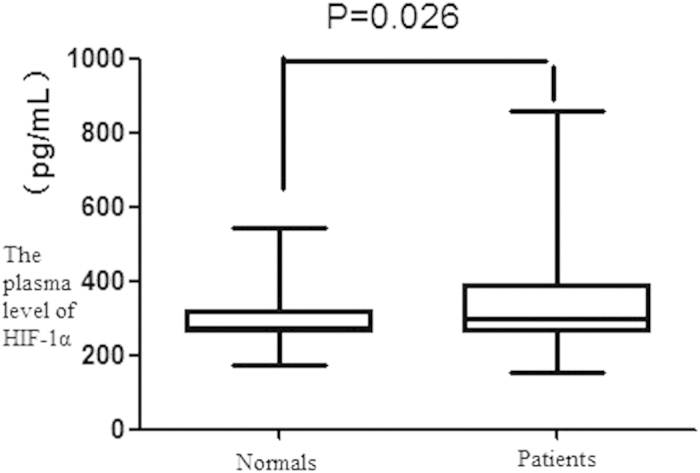
The plasma level of HIF-1α in NSCLC patients and normals. The plasma level of HIF-1α from NSCLC patients was higher than healthy volunteers, there was significant difference between two groups (P = 0.026).

The plasma level of HIF-1α of 100 cases of patients with NSCLC and 60 healthy volunteers were tested. The median levels of HIF-1α in plasma of patients with NSCLC and healthy volunteers were 297.70 pg/ml and 274.92 pg/ml respectively. The plasma level of HIF-1α from patients was higher than healthy volunteers’, there was significant difference between two groups (P = 0.026)(Fig. 1).

The relationship between the plasma level of HIF-1α and the clinical features (age, sex, histological type, tumor differentiation grade, T stage, local lymph node status, pTNM stage, tumor size and smoking condition) of patient with NSCLC was analyzed respectively. Statistically significant correlation was not found. The detailed data was shown in Table 3.

In this study, the plasma level of HIF-1α ≤ 297.70 was defined as a low level, the opposite as high level. The plasma levels of HIF-1α of patients with squamous carcinoma in different N stages was shown in Table 4. There was a significant difference between the two groups (X^2^ = 4.539, P = 0.033).

### The correlation between postoperative survival rate, clinicopathological features and the plasma level of HIF-1α of patients with NSCLC

By the end of the last follow-up time of this study (2014.11.30), follow-up has been available in all 100 cases. The median follow-up was 69.1 months (range: 3.4~79.7months). The 1-, 3- and 5-year postoperative survival rate of patients with NSCLC were 91%, 69%, 59% respectively, and not correlated with the plasma level of HIF-1α, age, gender, tumor differentiation grade, diameter of tumor and smoking status (P > 0.05). There was a significant relationship between the 3- and 5-year postoperative survival rate and pTNM stage (P < 0.05) (Table 5).

There was a significant relationship between 5-year postoperative survival rate of patients with squamous cell carcinomas and the plasma level of HIF-1α. The cut-off value of the plasma level of HIF-1α was set at the median (297.70 pg/ml). Patients with squamous cell carcinomas were divided into two groups (High Level > 297.70 pg/ml, Low level ≤ 297.70 pg/ml). The 5-year postoperative survival rate of low level group and high level was 78.6% (22/28), 50.0% (11/22) respectively. The difference between the two groups was statistical significantly (P = 0.034). There was no significant relationship between the 5-year postoperative survival rate and the plasma level of HIF-1α in patients with adenocarcinoma (Table 6).

### The overall survival curves analysis

**Figure 2:**
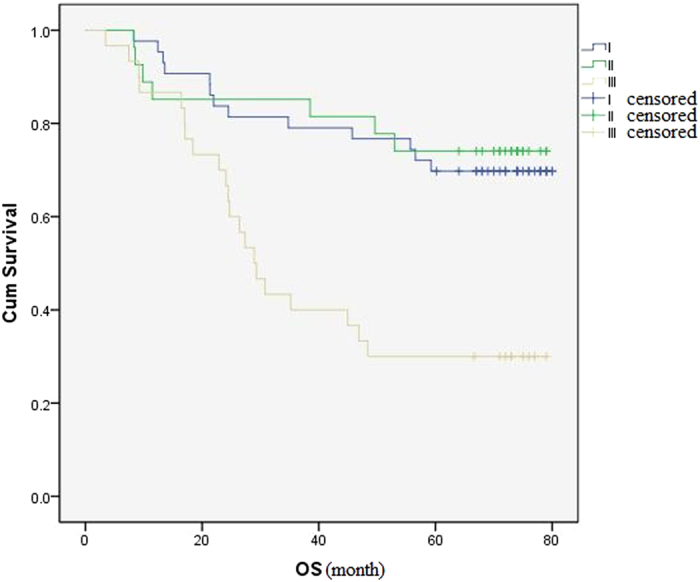
The differences between stage I, II, III. The overall survival rate of patients with stage I, II, III was 69.8%, 74.1%, 30.0% respectively. The differences between three groups were statistical significantly (P < 0.001).

**Figure 3:**
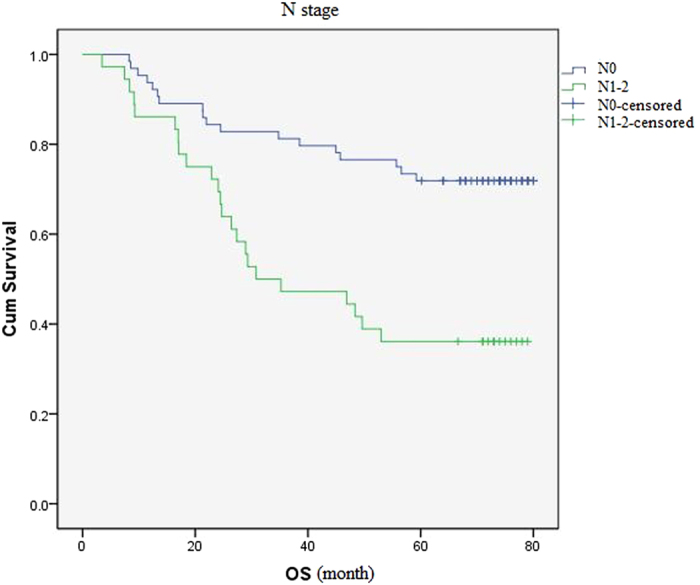
The overall survival rate of patients with/without regional lymph node metastasis. The overall survival rate of patients without regional lymph node metastasis was (71.9%), and significantly higher than patients had regional lymph node metastasis (P < 0.001).

A retrospective chart review was performed and the overall survival rate was estimated by using the Kaplan-Meier method and compared by Log-rank test. The results showed that: until 2014.11.30, the overall survival rate of patients with stage I, II, III was 69.8%, 74.1%, 30.0% respectively. The differences in three groups, significantly, were statistical (P < 0.001) (Fig. 2). The overall survival rate of patients without regional lymph node metastasis was 71.9%, and significantly higher than patients had regional lymph node metastasis (P < 0.001) (Fig. 3).

**Figure 4:**
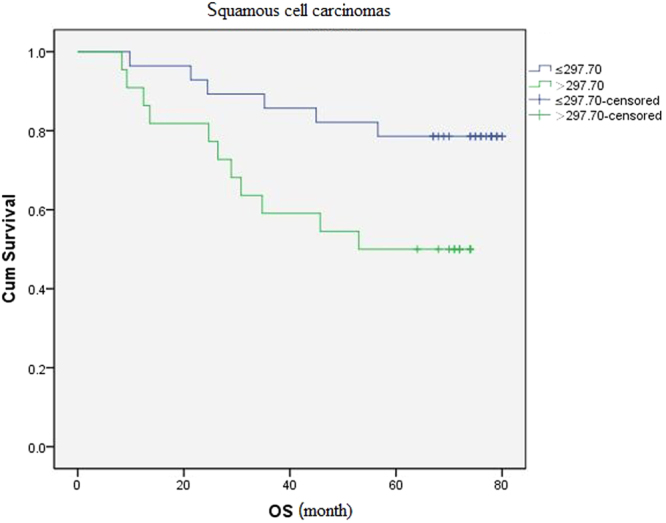
The overall survival rate of patients with squamous cell carcinomas with different plasma levels of HIF-1α. The difference between the two groups was statistical significantly (P = 0.028).

Patients were divided into two groups according to the plasma level of HIF-1α: the cut-off value was set at the median (297.70 pg/ml), in group I the plasma level of HIF-1α was ≤297.70/ml (Low group), and in group II was >297.70/ml (High group). The overall survival rate of patients with squamous cell carcinomas in low group and high group was 78.6%, 50% respectively. The difference among the two groups was statistical significantly (P = 0.028) (Fig. 4).

**Figure 5:**
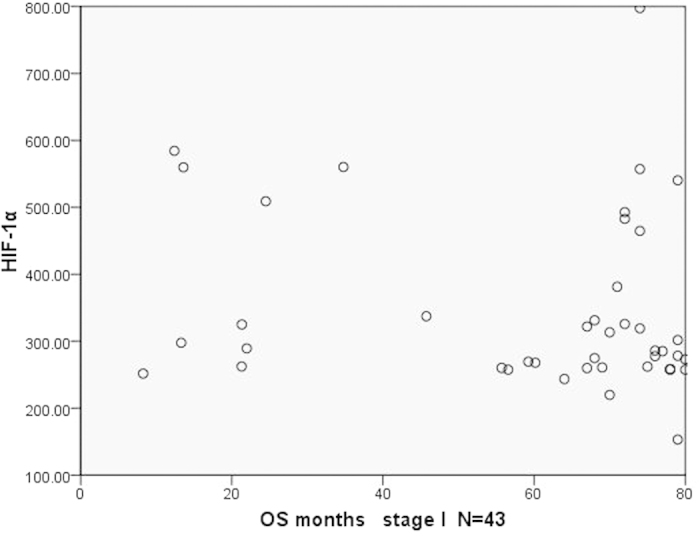
The scatter plot of OS and the plasma level of HIF-1α in patients (stage I).

**Figure 6:**
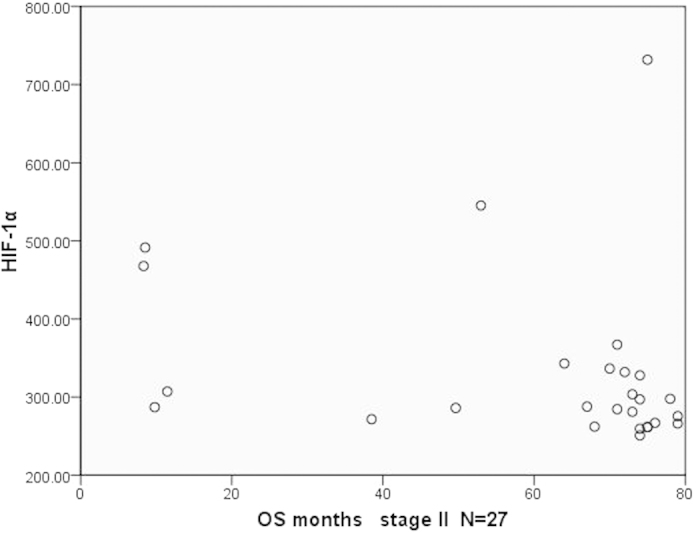
The scatter plot of OS and the plasma level of HIF-1α in patients (stage II).

**Figure 7:**
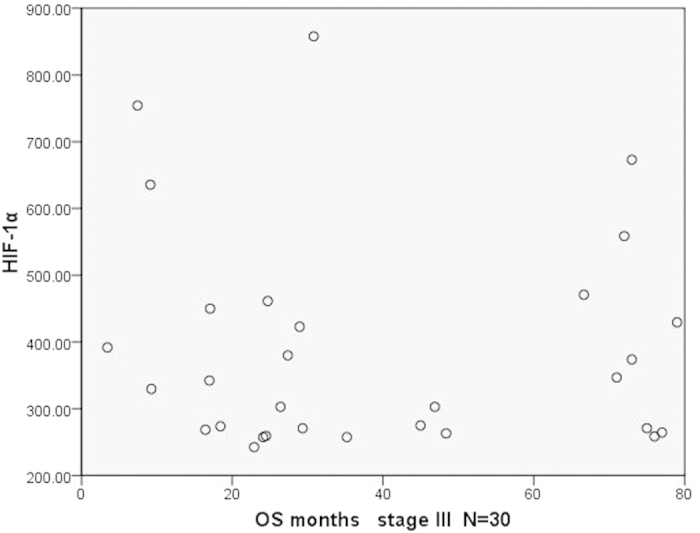
The scatter plot of OS and the plasma levels of HIF-1α in patients (stage III).

We further analyzed the relationship between the plasma HIF-1α and OS of patients in the same pTNM stage. Results showed that there was no statistical significance between both of them. To make the results more intuitive, we presented the results by a dot in a scatter plot (Figs 5, 6, 7). As we can see in the Figures, there was no statistical significance between both of them, but there were some trends in the mass. Patients with longer survival period were enriched in the lower half of the scatter plots. Therefore, if the sample size could be expanded in further studies, we may get results with statistical significance.

### Analysis of Prognostic Factors in NSCLC patients

By the end of this study, in 100 cases patients, 59% patients were survival. The median survival time of dead patients was 69.1 months (Range: 3.4~79.7months). The multivariate analysis of survival related factors was statistically analyzed by using Cox proportional hazard model. The results demonstrated that: a) Analysis of all patients’ independent prognostic factors. We showed that pTNM stage was a influential prognostic factor for overall survival of patients with NSCLC (HR: 1.895, 95% CI: 1.296–2.771, P = 0.001). The rest of clinical and pathological features were all not (P > 0.05). b) Analysis of independent prognostic factors in all patients with squamous cell carcinomas. pTNM stage (P = 0.002) and the plasma level of HIF-1α (P = 0.028) were independent prognostic factors of patients with squamous cell carcinomas. c) Analysis of independent prognostic factors in all patients with adenocarcinoma. The results had no statistical significance.

## Discussion

Because of the HIF-1α is related with hypoxia, this index has been widely used in the assessment of tumor hypoxia condition. But there is no unified and effective method to evaluate the activity level of HIF-1α within the tumor cells. Currently, immunohistochemistry (IHC) is the main research method whichwas used in the evaluation of HIF-1α levels in tumor patients. In addition, western bltting and reverse transcriptase polymerase chain reaction (RT-PCR) are also used in some researches. It has been reported that, for patients with psoriasis and require dialysis treatment, their angiogenesis and hypoxia condition can be evaluated by detecting the HIF-1α level within blood^34^,^35^. Additionally, there are limited prospective studies to evaluate the effect of breast cancer treatment by monitoring the HIF-1α level within the blood^36^. In China, some researches were reported that the treatment curative effect can be reflected by detecting the dynamic change of the HIF-1α level in patients with NSCLC and liver cancer before and after the interventional therapy^38^,^40^. But at present, reports about monitoring the level of HIF-1α within blood in patients with cancer are still rare.

Due to the imbalance of oxygen supply and oxygen consumption in the process of tumor growth, some hypoxic regions will subsequently appear, and further promote the rapid growth of cancer cells and the abnormal angiogenesis within tumors. Hypoxia is a common characteristic of solid tumors including NSCLC. Even in some tumor cells of early stage cancers, the condition of hypoxia is still evident. Many studies have since shown that the degree of hypoxia is related to insensitivity to chemotherapy and radiotherapy for patients with NSCLC, hihger invasive ability and a poor prognosis.

HIF-1α is the most crucial transcriptive factor that regulates diverse biological functions of a series of target genes such as proliferation, metastasis, invasion, apoptosis, and angiogenesis. Under the hypoxia environment, HIF-1α is activated in tumor cell, thus regulating the expression of hundreds of downstream target genes, thus promoting the activation of a large amount of protein factors, which participate in both tumor neovascularization and metastasis. It has been reported to be an important predictor of tumor progression for some types of solid tumors.

The aim of this study was to detect and analyse the plasma level of HIF-1α in patients with NSCLC, thus finding the relationships between the plasma level of HIF-1α and clinicopathological features. The survival data of postoperative patients with NSCLC was follow-up and collected. Correlation analysis of survival data, the plasma level of HIF-1α and clinicopathological features were analysed, and the results showed that the plasma level of HIF-1α was not an independent prognostic factor for patients with NSCLC. The pTNM stage was an independent prognostic factor for patients with NSCLC.

Currently, research on the level of HIF-1α of tumor patients is reported less. Some researches demonstrated that the plasma level of HIF-1α in patients with breast and liver cancer was significantly higher than normal person^37^,^38^. Dongping Xia *et al.* reported that the plasma level of HIF-1α in patients with NSCLC was significantly higher than healthy controls^39^. In this study, the plasma level of HIF-1α in patients with NSCLC was significantly higher than healthy volunteers’ (P = 0.026), and was consistent with the results of previous researches. But the results about the relationship between the plasma level of HIF-1α and tumor patients have not been reported yet. This research also didn’t find a significant relationship between the plasma level of HIF-1α and clinicopathological features of patients with NSCLC. The current researches and this study all found that the plasma level of HIF-1α of patients with NSCLC was higher than normal people’s. The reason might be tumor tissues with HIF-1α protein high expression appeared tissue necrosis, which resulted in a huge amount of HIF-1α entering the bloodstream, or there was a special regulation mechanism in hematological system itself of patients with NSCLC.

There are no reports about the plasma level of HIF-1α related to prognosis of patients with tumors at present. The correlation between both was first reported in this study. Although in the analysis of prognostic factors, the plasma level of HIF-1α was not a prognostic factor of patients with NSCLC, and was associated with the 5-year survival rate of patients with squamous cell carcinomas. In this study, the 5-year survival rate of patients with squamous cell carcinomas which their plasma level of HIF-1α lower than median was 78.6%, and much greater than the 5-year survival rate of those which the plasma level of HIF-1α higher than median (50.0%) (P = 0.034). Therefore, the plasma level of HIF-1α still can predict the prognosis of patients with NSCLC to some extent. In stratified analysis, we found that the plasma level of HIF-1α of patients with squamous cell carcinomas was associated with regional lymph node status.

In addition, there was a significant difference between different genders in the 3-year survival rate of patients with NSCLC and no statistically significant difference in 1-year survival rate of patients with different pTNM stages in this research. The reason may be related to the less number of cases and shorter follow-up time.

The results of this study demonstrated that HIF-1α may play crucial roles in some important processes of tumors, for example the growth progress and metastasis of the squamous cell carcinomas, or tumor angiogenesis, the plasma level of HIF-1α has crucial significance for the prediction of the prognosis of patients with NSCLC, especially in squamous cell carcinomas. However, it is still unclear that if the plasma level of HIF-1α was tested by dynamic monitoring, whether the results can reflect the curative effect of NSCLC related treatment, whether the plasma level of HIF-1α can reflect the condition of the patients’ disease. Previous studies had shown that many existing drugs, such as topoisomerase inhibitors-topotecan and etoposide had inhibition against HIF-1α^40^. It is still not clear that whether there will be a better curative effect when these drugs used in the patients of HIF-1α high expression. In addition, curative effects still need to be wait for the evaluation of the testing stage of new HIF-1α inhibitors^25^,^26^. Carry out more and further research on HIF-1α related maybe can find better HIF-1α target inhibitors and more appropriate patient group.

This study was designed to detect the plasma level of HIF-1α of 100 cases patients with NSCLC and the survival data was analyzed through follow-up. The results showed that the plasma level of HIF-1α of patients with NSCLC was significantly increased more than healthy people’s. The influencing factor of 3- and 5-year postoperative survival rate of patients with NSCLC was pTNM stage. No associations were observed between the plasma level of HIF-1α and the postoperative survival rate of patients, but the plasma level of HIF-1α was associated with the postoperative survival rate of patients with squamous cell carcinomas. The results of this study suggested that the plasma level of HIF-1α maybe provide a possible direction for further research and test, such as research on new therapeutic target of drugs based on HIF and the determination of drug-sensitive tumor types for HIF-1α inhibitors.

## Methods

In this study, in order to provide valuable reference for the research in the future, we detected the plasma level of HIF-1α and analyzed the relationship between the clinical pathological features of patients with NSCLC and the plasma level of HIF-1α.

### Ethics Statement

This study was approved by the Ethics Committee of Beijing Chest Hospital, Capital Medical University/Beijing Tuberculosis and Thoracic Tumor Research Institute. Written informed consent was obtained from each patient and healthy volunteer, and the acquisition of blood samples was carried out as prescribed by the institutional guidelines. The method described in this study was carried out in accordance with the approved guidelines and regulations.

### Patients and Healthy volunteers

In this study, patients who underwent pulmonary neoplasms resection (lobectomy or penumonecromy) in the thoracic surgery of Beijing Chest Hospital, Capital Medical University were recruited from May 2007 to August 2009. At last, a total of 100 patients were included in the study, the results of histopathological examination and different differentiation grades were based on the classification system of the World Health Organization revised in 2004 and the TNM staging system of UICC 2009 (Version 7). At the same time, 60 cases of healthy volunteers were recruited. The clinical characteristics of the 100 patients and 60 cases of healthy volunteers were presented in Table 1. The enter criterion of this study was presented in Table 7.

The preoperative blood samples were collected from all the patients enrolled in this study, for the detection of plasma level of HIF-1α. At the same time, the blood samples of 60 cases of healthy volunteers were collected and detected too.

Collecting and dealing with the samples: 5 ml of fasting blood sample were drawn from antecubital vein of every patient with NSCLC in the morning 1–2 days before operation. The blood samples were put into the EDTA anticoagulative tube, then centrifuging ten minutes (1000 r/min) 2 hours after drawing blood, separated plasma, stored in −80 °C to be determined. Vein blood samples were collected with limosis from healthy volunteers at the same period, the same process method.

### HIF-1α Enzyme-Linked Immunosorbent Assay (ELISA)

The concentration of HIF-1α in the NSCLC patients and healthy volunteers were determined using ELISA regent kits (SINO-AMERICAN BIOTECHNOLOGY CO. LTD. Luoyang, China) according to the manufacturer’s instructions and analyzed using a Labsytems Multiscan reader. The experiment was repeated twice with triplicate measurements in each experiment (measuring the optical density of each well at 450 nm wavelength within 5 minutes at the end of reaction).

### Follow-up

All patients were followed-up by phone call consultation every 3 to 6 months after they were discharged from the hospital. Follow-up was completed in all patients until 2014.11.30 or the patient dead, and the median follow-up period was 68.5 months (range: 3.4~79.7months). During the period of follow-up, 1 case of patients was excluded because of dead within 3 months. In the rest of the 100 patients, all cases completed 5 years of follow-up. The follow-up time was censored if the patient was lost or dead during follow-up.

### Statistical Analyses

All statistical analyses were examined using SPSS19.0 statistical software. Non-normal distribution was represented by the median (M). X^2^ test was used to analyze the relationship between two categorical variables. In this study, comparison between patients and healthy volunteers; correlation between the clinical characteristics of NSCLC patients; comparison between the plasma level of HIF-1α related to the N stage of patients with squamous cancer; the single-factor analysis of the 1-, 3- and 5-year postoperative survival rate for patients; the univariate analyses of 5-year survival rate of adenocarcinoma and squamous carcinoma patients were all analysed by using X^2^ test. When the sample size is less than 5, Fisher’s exact test was used.

The differences between two measuring parameters were analyzed by Mann-Whitney U test. In this study, the relationship between the clinical features and plasma level of HIF-1α, age of patients, comparison of the plasma level of HIF-1α between patients and healthy volunteers were analyzed by Mann-Whitney U test. Kruskal-Wallis H test was used between three or more measuring parameters.

Overall survival was assessed by the Kaplan–Meier method, while log rank test was used for comparison. Prognostic factors of OS were analysed by univariate and multivariate analysis. Variables with *P* < 0.05 and potential clinical confounding effects (e.g. age, sex and stage) were added to the final multivariate Cox proportional hazard model. Survival curves were drawn using the Kaplan-Meier method and compared by the log-rank test.

The comparison diagram of the plasma level of HIF-1α of NSCLC patients and healthy volunteers; comparison charts of the plasma level of HIF-1α associated with squamous carcinoma N stage were drawn by Graphpad Prism made 5.

## Additional Information

**How to cite this article**: He, J. *et al.* The relationship between the preoperative plasma level of HIF-1α and clinic pathological features, prognosis in non-small cell lung cancer. *Sci. Rep.* **6**, 20586; doi: 10.1038/srep20586 (2016).

## Figures and Tables

**Table 1:** The clinical characteristics of the 100 patients and 60 cases of healthy volunteers.

clinical characteristics	Case of patients(%)	healthy volunteer	X^2^	P
Age				
Median	59	57		0.432
Range	36–84	31–82		
Gender				
Male	80(80%)	44(73%)	0.361	0.665
Female	20(20%)	16(27%)		
Pathological type				
Adenocarcinoma	40(40%)			
Squamous cell carcinomas	50(50%)			
Others	10(10%)			
Tumor differentiation grade				
Poorly differentiated	44(44%)			
Middle differentiated	55(55%)			
High differentiated	1(1%)			
pTNM stage				
Ia stage	20(20%)			
Ib stage	23(23%)			
IIa stage	19(19%)			
IIb stage	8(8%)			
IIIa stage	30(30%)			
N stage				
N0	64(64%)			
N1	9(9%)			
N2	27(27%)			
Smoking				
Yes	59(59%)			
No	41(41%)			

**Table 2:** Correlation analysis between the clinical features of NSCLC patients.

	Pathological type		X^2^	P	Tumor Size		X^2^	P	Smoking		X^2^	P
	Adenocarcinoma (n, %)	Squamous cell carcinomas (n, %)			Small (n, %)	Big (n, %)			Yes (n, %)	No (n, %)		
Gender												
Male	25(34.2)	48(65.8)	16.278	<0.001	43(53.8)	37(46.3)	4.558	0.33	59(73.8)	21(26.3)	35.976	<0.001
Female	15(88.2)	2(11.8)			16(80.0)	4(20.0)			0(0)	20(100)		
Smoking												
Yes	16(29.1)	39(70.9)	13.502	<0.001	30(50.8)	29(49.2)	3.954	0.047				
No	24(68.6)	11(31.4)			29(70.7)	12(29.3)						
Diameter of tumor												
≤4cm	31(56.4)	24(43.6)	8.137	0.004								
>4cm	9(25.7)	26(74.3)										

**Table 3:** The relationship between the plasma level of HIF-1α and the clinical features.

	Cases	the plasma level of HIF-1α(pg/ml)	P
		Median(Range)	
Age			
≤60y	56	286.43(242.34–857.89)	0.268
>60y	44	322.06(153.04–754.30)	
Gender			
Male	80	297.70(153.04–857.89)	0.670
Female	20	288.14(243.62–754.30)	
Pathological type			
Adenocarcinoma	40	305.27(153.04–754.30)	0.573
Squamous cell carcinomas	50	286.02(219.84–857.89)	
Others	10	272.09(242.34–635.60)	
Tumor differentiation grade			
Poorly differentiated	44	302.25(242.34–754.30)	0.397
Middle-, high-differentiated	56	286.43(153.04–857.89)	
T stage			
T1	30	320.55(257.46–754.30)	0.495
T2	59	286.94(153.04–857.89)	
T3-4	11	307.70(250.81–558.71)	
N stage			
N0	64	285.51(153.04–797.68)	0.462
N1-2	36	330.74(242.34–857.89)	
pTNM stage			
I	43	285.92(153.04–797.68)	0.467
II	27	287.68(250.81–731.88)	
III	31	335.92(242.34–857.89)	
Diameter of tumor			
≤4cm	59	302.77(153.04–857.89)	0.516
>4cm	42	285.92(219.84–757.68)	
Smoking			
Yes	59	302.76(219.84–857.89)	0.343
No	41	285.92(153.04–754.30)	

**Table 4:** The plasma level of HIF-1α of patients with squamous cell carcinoma in different N stages.

	Low level (n, %)	High level (n, %)	Total
N0	24(64.9%)	13(35.1%)	37
N1-2	4(30.8%)	9(69.2%)	13
Total	28	22	50

X^2^ = 4.539, P = 0.033.

**Table 5:** Mono-factor analysis of postoperative survival rate of patients with NSCLC.

	1-year postoperative survival rate(%)	P	3-year postoperative survival rate(%)	P	5-year postoperative survival rate(%)	P
Age						
≤60y	91.1%	0.978	67.9%	0.780	64.3%	0.225
>60y	90.9%		70.5%		52.3%	
Gender						
Male	93.8%	0.055	73.8%	0.040	62.5%	0.155
Female	80.0%		50.0%		45.0%	
Pathological type						
Adenocarcinoma	92.5%	0.777	72.5%	0.873	57.5%	0.409
Squamous cell carcinomas	94.0%		74.0%		66.0%	
Tumor differentiation grade						
Poorly differentiated	88.6%	0.464	63.6%	0.304	54.5%	0.422
Middle-, high-differentiated	92.9%		73.2%		62.5%	
Diameter of tumor						
≤4cm	93.2%	0.352	67.8%	0.755	61.0%	0.623
>4cm	87.8%		70.7%		56.1%	
pTNM stage						
I stage	97.7%	0.126	79.1%	<0.001	69.8%	0.001
II stage	85.2%		85.2%		74.1%	
III stage	86.7%		40.0%		30.0%	
Smoking status						
Yes	93.2%	0.352	74.6%	0.148	62.7%	0.365
No	87.8%		61.0%		53.7%	
The plasma level of HIF-1α						
≤297.70 pg/ml	96.2%	0.061	76.9%	0.075	63.5%	0.345
>297.70 pg/ml	85.4%		60.4%		54.2%	

**Table 6:** Mono-factor analysis of 5-year postoperative survival rate of patients with different pathological type.

	5-year postoperative survival rate of Squamous cell carcinomas	P	5-year postoperative survival rate of Adenocarcinoma	P
Age				
≤60y	68.8%	0.584	70.6%	0.150
>60y	61.1%		47.8%	
Gender				
Male	66.7%	0.626	60.0%	0.680
Female	50.0%		53.3%	
Tumor differentiation grade				
Poorly differentiated	60.0%	0.091	46.2%	0.314
Middle-, high-differentiated	72.0%		63.0%	
Diameter of tumor				
≤4cm	66.7%	0.778	61.3%	0.368
>4cm	65.4%		44.4%	
pTNM stage				
I stage	75.0%	0.001	68.8%	0.342
II stage	82.4%		62.5%	
III stage	11.1%		43.8%	
Smoking status				
Yes	66.7%	0.851	56.3%	0.896
No	63.6%		58.3%	
The plasma level of HIF-1α				
≤297.70 pg/ml	78.6%	0.034	47.1%	0.251
>297.70 pg/ml	50.0%		65.2%	

**Table 7:** The enter criterion of this study.

Research object	Healthy volunteer
1. Stage Ia-IIIa NSCLC patients diagnosed by cytology or pathology preoperatively; or confirmed by surgery pathology	1. Over the age of 18
2. Preoperative routine examination and functional evaluation are in line with the operation indication	2. No diseases of heart, liver, lungs and kidneys and other vital organs.
3. The surgery method is radical resection of the primary lung tumor	3. The collection time of female can’t in menstrual period and pregnancy period.
4. Patients did not receive preoperative chemotherapy, radiotherapy, tumor targeted therapy, biological treatment and any other tumor-related treatment	
5. No injuries and other surgery within the last two months	
6. No history of other cancers	
7. No diseases of heart, liver, lungs and kidneys and other vital organs	
